# Bisphosphonate Therapy and Tooth Development in Children and Adolescents with Osteogenesis Imperfecta

**DOI:** 10.1007/s00223-020-00707-1

**Published:** 2020-05-25

**Authors:** Barbro Malmgren, Georgios Tsilingaridis, Nina Monsef-Johansson, Zaina Haif Al Qahtani, Göran Dahllöf, Eva Åström

**Affiliations:** 1 Department of Dental Medicine, Division of Orthodontics and Pediatric Dentistry, Karolinska Institutet, Huddinge, Sweden; 2 Center for Pediatric Oral Health Research, Stockholm, Sweden; 3 Department of Woman and Child Health, Karolinska Institutet, Stockholm, Sweden; 4 Pediatric Neurology, Astrid Lindgren Children’s Hospital at Karolinska University Hospital, Stockholm, Sweden

**Keywords:** Bisphosphonate, Osteogenesis imperfecta, Tooth development, Bone resorption, Dental age, Root resorption

## Abstract

Osteogenesis imperfecta (OI) is a heterogeneous connective tissue disorder characterized by repeated fractures and skeletal disorders. At present, bisphosphonate (BP) therapy is the gold standard for OI treatment. The present retrospective study evaluated the effect of BP therapy on tooth development and eruption of permanent teeth in a cohort of children receiving pamidronate. Three groups were studied: patients with OI who were treated with BPs (*n* = 45), patients with OI who were not treated with BPs (*n* = 117), and age- and gender-matched healthy controls (*n* = 121). Dental age, dental maturity, and tooth eruption were assessed on panoramic radiographs using the methods of Demirjian et al. (Hum Biol 45(2):211–227, 1973) and Haavikko (Suom Hammaslaak Toim 66(3):103–170, 1970) and were evaluated using the *t*-test, Chi-square test, and the Mann–Whitney *U* test. Dental age in the study group was significantly (*p* < 0.05) lower than chronological age compared with both control groups. Dental maturity and the eruption of permanent teeth were also significantly (*p* < 0.05) delayed in the study group in relation to the two control groups. The dental age was significantly lower (*p* < 0.001) in patients with OI type III treated with BPs compared with healthy controls and the dental maturation was significantly delayed in patients with OI type IV treated with BPs compared with those not treated. In conclusion, BP therapy in OI patients seems to lower the dental age, delay the dental maturity, and tooth eruption. BP administration before 2 years of age might be a contributing factor.

## Introduction

Osteogenesis imperfecta (OI) is a heterogeneous connective tissue disorder [1].The disease is usually inherited in an autosomal dominant manner, although new mutations are common. In 85–90% of the cases, the mutations are located in the *COL1A1* and *COL1A2* genes in chromosomes 17 and 7, respectively [2]. In recent years, several other genes have been associated with the disease [3], and there are now more than 1500 known mutations in over 20 different genes. New mutations are being detected and reported over time, and their effects on tissue structure and biomechanics studied [4–6]. The clinical manifestations may include bone fragility, skeletal deformities, growth and hearing deficiencies, joint instability due to laxity of ligaments, coagulation and bleeding disturbances, skeletal pain, blue sclerae, dentinogenesis imperfecta (DGI), and other dental development disorders [2, 3, 5, 7–10], of which tooth agenesis is the most frequent [11]. The most commonly used classification of OI is based on clinical, radiological, and hereditary findings: type I (mild OI with blue sclera), type II (pre- or perinatal lethal), type III (severely deforming), and type IV (variable but often moderate severity, and white sclerae after infancy) [10].

The core feature of OI therapy is fracture prevention. Treatment with bisphosphonates (BPs) is today the gold standard treatment for children with OI [12]. Alendronate, risedronate, pamidronate, and zoledronate are today the most commonly bisphosphonates used for treatment in individuals with OI although in children intravenous pamidronate or zoledronate are most frequently used. They act mainly by inhibiting osteoclast function. Intravenous BPs have proven to decrease bone pain and improve muscle strength and mobility. It increases cortical thickness, vertebral size and architecture and bone mineral density and thereby decreased fracture incidence [13–17]. Treatment with pamidronate infusions started 1991 in Sweden and it was initially given to adolescents with severe OI but later also to younger children with the more severe phenotypes of OI, types III and IV. Later on, it was also given to individuals with milder phenotype but with vertebral compressions, repeated bone fractures, and pain. Before 1991 treatment with physiotherapy, orthotics, orthopedic surgery, and intravenous calcitonin were the only available treatment options [14].

Tooth eruption involves bone resorption and deposition [18], and it is well known that osteoclasts are important for tooth eruption and for resorption of the primary teeth [19]. Two human studies [20, 21] on how BP therapy affects tooth development reported conflicting results. Kamoun-Goldrat et al. [20] claimed that the therapy was associated with delayed tooth eruption, while Vourimies et al. [21] found age-appropriate dental development.

The present study evaluated the effect of BP therapy on the maturation and eruption of permanent teeth in a cohort of 45 children and adolescents with OI, whose ages ranged from newborn to 12 years at the start of therapy, and who had undergone treatment for at least 1 year. Healthy children and adolescents and children and adolescents with OI who had not had treatment with BPs acted as controls. The hypotheses were that BP treatment delays dental maturity, dental age, and tooth eruption when treatment begins in early childhood.

## Methods

### Subjects

All participants with OI had received care at Astrid Lindgren Children’s Hospital from the national multidisciplinary pediatric OI team at Karolinska University Hospital in Stockholm between 1991 and 2018. Fifty-three children and adolescents with OI (26 boys and 27 girls) treated with BPs and 121 children with OI (76 boys and 46 girls) who had not been treated with BPs were identified. The 126 healthy age- and gender-matched children and adolescents had been patients at the Department of Pediatric Dentistry at Karolinska Institutet. All OI patients and healthy controls who were under age 16 years at the time of their radiographic examination were included. Excluded were individuals who were missing both lower second premolars: two boys and six girls with OI treated with BPs, four boys and one girl with OI not treated with BPs, and five healthy controls (three boys and two girls). Of the excluded children treated with BPs, both boys and five of the six girls had begun BP treatment before 2 years of age.

The included participants formed three groups: those with OI who had been treated with BPs (the study group); those with OI who had not been treated with BPs (control group 1); and healthy, matched controls (control group 2).

*The study group* consisted of 45 children and adolescents with OI, 24 boys and 21 girls treated with BPs for at least 1 year. In 14 children, seven boys and seven girls, BP therapy was begun before the age of 2 years. Data extracted from existing dental and medical records included panoramic radiographs, medication, and type of OI. Nineteen children presented with OI type I, 12 with OI type III, and 14 with OI type IV. The mean age at radiographic evaluation was 10.1 years (5.7–15.0). In 30 subjects, BP therapy started before 6 years of age and in 14 from 6 up to 12 years. Only one individual had started BP therapy after 12 years. The gender distribution was equal with 16 boys and 14 girls in the first group, and seven boys and seven girls in second.

The extracted data on BP therapy included doses, duration, and cumulative dose. Mean age at treatment start was 3.9 years (0.2–13.0) The infusions were initially given monthly in doses of 10–30 (40) mg/m^2^ pamidronate during 5–8 h preceded by hydration with 25 mg/ml of buffered glucose (total dose of 500 ml/m^2^ for 2–4 h). For the first 3 months, a dose of 10 mg/m^2^ was given, over the next 3 months 20 mg/m^2^, followed by 30 mg/m^2^ for further treatment. After these first 6 months, a few patients received a dose of 40 mg⁄m^2^. Treatment time was later shortened to 4 h without pre-treatment hydration. After normalization of bone mineral density, treatment intervals were individualized, successively increasing to once every 2–6 months depending on bone density values and pain.

*Control Group 1* included 117 children with OI, 72 boys and 45 girls who had not been treated with BPs. Data were extracted from existing dental and medical records including panoramic radiographs, medication, and type of OI. Ninety-six children presented with OI type I, 2 with OI type III, and 19 with OI type IV. Children and adolescents with severe forms of OI born before BPs were introduced as a therapy was included in this group.

*Control Group 2* consisted of 121 randomly selected healthy age- and gender-matched children and adolescents, 55 boys and 66 girls, from the Department of Pediatric Dentistry at Karolinska Institutet. Data were extracted from existing panoramic radiographs taken between 2016 and 2018. Inclusion criteria were healthy children aged 4–16 years.

Table 1 describes the characteristics of the study group and the two control groups.

**Table 1:** Group characteristics of the children and adolescents with osteogenesis imperfecta (OI), with and without bisphosphonate (BP) treatment, and healthy age- and gender-matched controls

	OI type I	OI type III	OI type IV	Total
*Study group: with OI, BP treatment*				
Number of subjects	19	12	14	45
Gender (M/F)	11/8	4/8	9/5	24/21
DGI (yes/no)	4/15	5/7	5/9	14/31
Age at imaging	10.5 (6.0–15.0)	9.6 (5.7–13,8)	9.8 (7.6–14.1)	10.1 (5.7–15.0)
Age at treatment onset	5.4 (0.25–10.4)	1.9 (0.3–7.1)	4.9 (0.2–12.5)	3.5 (0.2–12.5)
Treatment, years^a^	5.2 (0.9–13.0)	7.7 (4.3–12.8)	5.1 (1.0–11.0)	5.8 (1.0–13.0)
Cumulative BP dose (mg/m^2^)	1097 (260–2180)	1333 (710–2730)	1138 (240–3850)	1189 (240–3850)
*Control group 1: with OI, no BP treatment*				
Number of subjects	96	2	19	117
Gender (M/F)	59/37	1/1	12/7	72/45
DGI (yes/no)	8/88	2/0	7/12	16/101
Age at imaging	9.4 (4.1–15–8)	11.6 (10.1–13.1)	9.1 (6.3–14.2)	9.4 (4.3–15.8)
*Control group 2: Healthy controls*				
Number of subjects				121
Gender (M/F)				55/66
DGI (yes/no)				–
Age at imaging				8.6 (4.2–15.4)

^a^From treatment onset to study start

*DGI* Dentinogenesis imperfecta

The regional ethics committee in Stockholm approved the study protocol (Daybook no. 157/99 and 2014/254-31/4).

### Radiographic Analyses

Conventional screen-film (*n* = 57) or digital panoramic radiographs (*n* = 226) from the dental records of all study participants were analyzed regarding permanent tooth development and number of erupted permanent teeth. The radiographs were anonymized, coded, and analyzed under standardized terms. A Mattsson’s binocular and a light table were used to assess the screen-film radiographs.

Two observers, NMJ and ZHQ, evaluated all radiographs from the three groups separately. BM and GT made a second determination independently. When more than one panoramic radiograph was available, the one with the best quality taken at age 9–10 years was chosen. When the scores differed, one of the two dentists (BM or GT) re-evaluated the radiograph.

Dental age and dental maturity were assessed by evaluating the mineralization stages of the seven mandibular teeth on the left side. The mineralization stages ranged from beginning of crown calcification to a completely closed root apex, as described by Demirjian et al. [22]. Each tooth was divided into eight formative stages (A to H), and each stage was allocated a score depending on the gender. If an assessment was between two stages, the earlier stage was selected [22]. If a tooth was missing, the contralateral tooth was analyzed.

The stages were converted to dental maturity scores and summarized. A maturity score was then derived from the addition of the scores of all seven teeth. This maturity score was converted directly into a dental age by using a pre-constructed table and the difference between the chronological and dental age was calculated.

Permanent tooth eruption was assessed for each patient. If the primary tooth was missing or had been extracted before expected exfoliation due to caries or infection, the contralateral tooth was analyzed. A tooth was recorded as erupted if it had penetrated the marginal bone level. We used the reference values for timing of tooth eruption from the Haavikko study on healthy Finnish children [23].

### Statistical Analysis

Between-group comparisons of dental scores, cumulative doses, and impact of DGI on eruption were done with the Mann–Whitney *U* test. The *t*-test assessed differences between chronological age and estimated dental age. Chi-square test was used to evaluate the number of erupted permanent teeth in each group compared with reported normal values [24]. All comparisons were made both according to gender and pooled. The level of significance was set at 5%. No comparisons could be made separately for the group “OI type III not treated with BP” since there were only 2 individuals.

We randomly selected 29 patients from the entire study sample to evaluate precision in estimating dental age according to Demirjian [22], and the number of erupted teeth with double determination according to the reference values of Haavikko [23]. Kappa values were used for inter-observer agreement. For dental scores, agreement was 90% (*ĸ* = 0.80), and for eruption, 86% (*ĸ* = 0.72), indicating a strong level of agreement.

All data were analyzed with Statistica v. 13 (StatSoft; Scandinavia AB, Uppsala, Sweden).

## Results

### Severity of OI

The cumulative BP dose was significantly higher in patients with OI type III compared with OI type I (*p* = 0.011) and IV (*p* = 0.014). There was no difference in doses between OI types I and IV.

The dental age was significantly lower in patients with OI type III treated with BPs compared with the healthy controls (*p* < 0.001), and dental maturity was significantly delayed in patients with OI type III (*p* < 0.001) and OI type IV (*p* = 0.024) treated with BP compared with the two control groups. No difference was found between boys and girls.

In patients with OI type I treated with BPs, the eruption was significantly (*p* = 0.034) earlier compared with healthy controls. The variation in number of erupted teeth was higher in the healthy controls (− 2 to + 4) than in OI type I (− 1.0 to + 2). No difference was found between boys and girls.

We found that the cumulative dose of BPs was significantly higher in patients were BP therapy started before 6 years of age compared with those starting the BP therapy from 6 up to 12 years (*p* < 0.001). Despite the higher cumulative dose in patients < 6 years of age, no differences in dental age, dental maturation or in eruption were found.

### Dental Age/Dental Maturity

Estimated dental age was significantly lower than chronological age in the study group compared to the two control groups: those not treated with BPs (*p* = 0.036) and the healthy controls (*p* = 0.032). Differences between the two control groups were not significant; neither were differences between individuals with and without DGI nor between boys and girls.

**Fig. 1:**
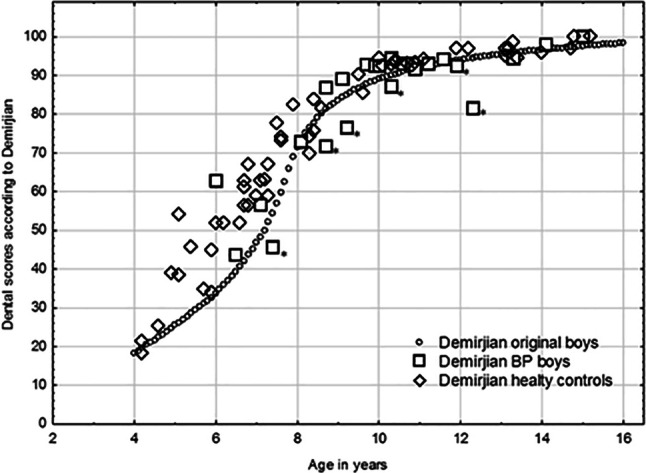
A scatterplot showing the dental scores according to Demirjian for 24 boys treated with BPs and 55 healthy controls. *Boys treated with BPs with a cumulative dose greater than 1000 mg⁄m^2^. *BPs* bisphosphonates

**Fig. 2:**
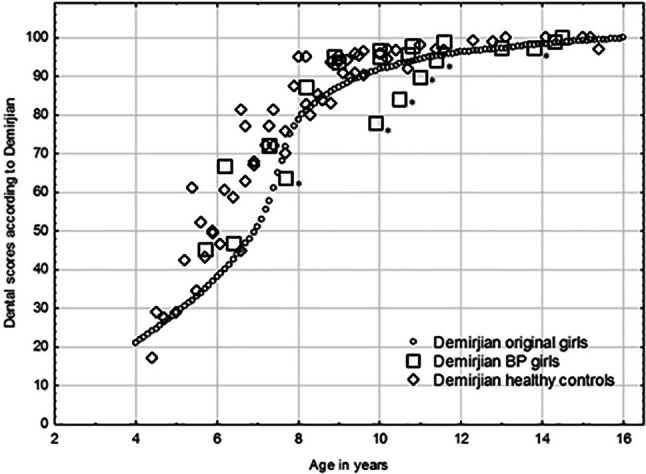
A scatterplot showing the dental scores according to Demirjian for 21 girls treated with BPs and 66 healthy controls. *Girls treated with BPs with a cumulative dose greater than 1000 mg⁄m^2^. *BPs* bisphosphonates

Dental maturity was significantly delayed in the study group compared with those not treated (*p* = 0.004) and to healthy controls (*p* = 0.002). Dental maturity was significantly delayed in boys with OI who had been treated with BPs compared with those who had not (*p* = 0.021), and also compared with healthy controls (*p* = 0.012). Differences similar to those for boys existed among girls, but the differences were not significant; between the study group and the OI group that had not been treated with BPs (*p* = 0.075), and between the study group and the healthy controls (*p* = 0.099; Figs. 1 and 2). Differences between the two control groups for both genders were non-significant (*p* = 0.917).

### Eruption

**Fig. 3:**
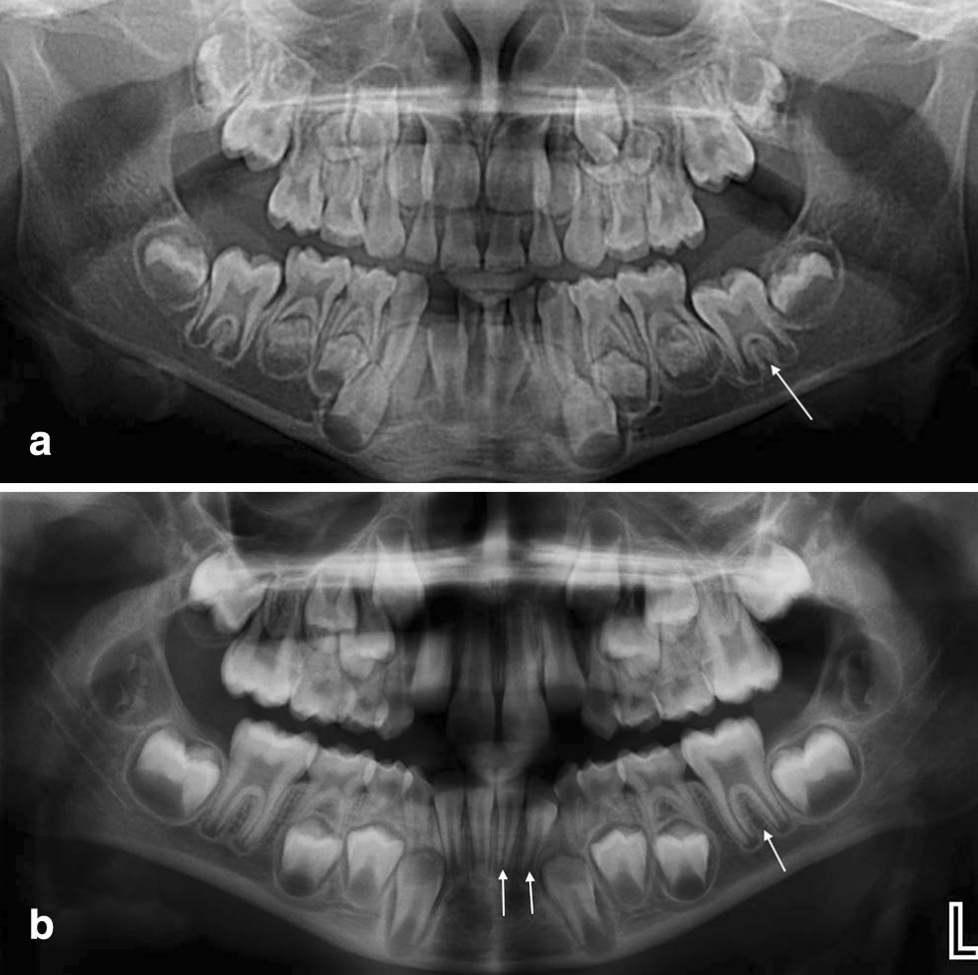
Two boys at 7.4 years of age. **a** treated with BP since the age 0.3 years, Demirjian dental score 45.6. Only the permanent lower left molar in the left mandibular quadrant erupted. **b** healthy control, Demirjian dental score 77.7. Three permanent teeth fully erupted in the left lower side. *BP* bisphosphonate

Eruption was significantly delayed in children with OI who had been treated with BPs, compared with those who had not (*p* = 0.001) and healthy controls (*p* = 0.008). Among boys with OI, eruption was significantly delayed in those who had been treated with BPs compared with those who had not (*p* = 0.037) and healthy controls (*p* = 0.030; Fig. 3). No significant difference was found in girls with OI who had been treated with BPs compared with those who had not (*p* = 0.547) and healthy control (*p* = 0.428). The average delay in boys was 1.5 years (− 0.1 to + 4.0), and in girls, 0.8 years (0 to + 3.4). No significant difference was found between the two control groups. There was no difference in number of erupted teeth in patients with and without DGI.

Dentinogenesis Imperfecta (DGI) was found in 21% of the patients with OI type I treated with BP and in 8% in those not. The gender distribution was equal. In patients with OI type III/IV, DGI was found in 38% of the patients both for those treated with BPs and for those not. Boys were more affected than girls.

## Discussion

The main findings in the present study are that the dental age and dental maturity of individuals treated with BPs are significantly lower when compared to patients with OI who had not been treated with BPs and healthy controls. Furthermore, tooth eruption was significantly delayed in children treated with BPs. In both control groups, maturity was accelerated in correlation with Demirjian’s dental scores [22]; but in contrast to Vuorimies et al. [21], we found no difference in dental maturity between the two control groups. When analyzing the different types of OI, we found that the dental maturation was significantly delayed in patients with OI types III and IV compared with healthy controls.

The system used to assess dental age in this study was the worldwide used assessment system of Demirjian et al. [22]. In many countries, dental maturity of Caucasian children has been found to be earlier than in the Demirjian assessment system [25–29], and dental age might be overestimated [30]. In 1998, a report described a trend towards earlier dental maturation in orthodontic patients between the 1970s and 1990s [31]. Another study showed that root maturation in children in a modern sample was advanced compared with children in a historic sample of 50 years before [32]. Jääsaari et al. [33] found that dental maturity of Finnish 6–12-year-old children was advanced by 0.6–1.0 years compared with chronological age and suggested that the advancement in dental maturity might be associated with higher energy intake. Earlier puberty has also been observed in girls and boys over the last century. The reasons for these changes are unknown but may be explained by environmental and social factors [34]. Genetic, hormonal, and environmental interactions as well as epigenetic and nutritional factors may also be involved in dental development.

We found that individuals treated with BPs demonstrated a significantly lower dental age and dental maturity when compared with OI patients not treated with BPs and healthy controls. This is in contrast to Vourimies et al. [21], who found no significance difference between chronological and dental age in their study group (only five subjects had been treated with BPs before the age of 2 years). One explanation for our results might be the higher number of children below 2 years of age who had been treated with BPs in the present study.

At present, the nitrogen-containing BPs pamidronate and zoledronate are the most commonly used for intravenous medication of children with OI. They have different in vitro potencies, with pamidronate having the lowest relative potency of 100, and zoledronic acid, the highest with a relative potency 10,000. In our study, only intravenous pamidronate was administrated. In the two human studies of Kamoun-Goldrat et al. [20] and Vourimies et al. [21], the age of the population, medical treatment, evaluation methods, and results differed. The ages of the 33 children and adolescents in the Kamoun-Goldrat et al. study [20] ranged from 6.2 to 14.6 years at the start of BP therapy; the researchers did not report which BP was being used. The ages of the 22 children and adolescents in the Vourimies et al. study [21] ranged from 0.1 to 13.0 years at the start of therapy; this research group used various types of BPs such as intravenous pamidronate and zoledronic acid, and oral administration of risedronate.

Resorption of the roots of primary teeth is needed for a permanent tooth to erupt. For that process to be possible, alveolar bone resorption must occur and a biological process for the tooth to move in the pathway must be possible [19]. BPs act by inhibiting osteoclast function and thereby decrease bone resorption. This effect on bone density persists for years since the half-life of BPs is a decade [2]. Since tooth eruption involves bone resorption and deposition [18, 35], osteoclasts are important for tooth eruption and for resorption of primary teeth [19]. BP-induced decreases in osteoclast activity may affect tooth development. We assessed eruption based on the results of Haavikko [23] because we used the same criteria for eruption in the panoramic radiographs. This study was published as early as 1970. Parner et al. [36] concluded that the emergence of permanent teeth was not subject to acceleration between 1969 and 1996 and, consequently, the study by Haavikko is still relevant. Parner et al. recorded a tooth as erupted when any part of it had emerged through the gingiva. Vuorimies et al. [21] related the number of erupted teeth to the study by Nyström et al. [37]. In their study, they recorded a tooth as erupted when any part of it had emerged through the gingiva while Vourimies et al. [21] recorded it as erupted also when it had radiographically pierced the alveolar bone, which makes a correlation unsure. In the present study, we demonstrate a significantly delayed tooth eruption in children with OI who had been treated with BPs compared with those who had not and healthy controls. We also found a significantly delayed tooth eruption among boys with OI treated with BP, compared the two control groups, which was not seen among girls.

Regarding eruption, Kamoun-Goldrat et al. [20] examined their subjects only by visual inspection of all four quadrants and the number of delayed teeth was determined using the classical data of Hurme [41] where a tooth was described as delayed if it had not emerged in the age range given by Hurme. Although they did not report type of OI, we assume that the majority had OI type III as they had been treated with BPs. We reported earlier [11] that tooth agenesis occurs more often in OI type III-affected individuals, 47%, compared with OI types I (12%) and IV (13%). Thus, as Vuorimies et al. [21] also discussed, the effect on tooth eruption in patients who had received BP therapy is likely overestimated. Vourimies et al. studied only the left mandibular teeth and used as reference values published norms for the timing of tooth eruption [37]. Our results differed from the results reported by Vourimies et al. One explanation might the higher number of individuals in all groups in our study. Furthermore, 14 children in the present study had started BP therapy before the age of 2 years compared to five in the former (personal communication).

The prevalence of DGI in OI type I varies from 8 to 40% [7, 8, 38–40]. In the present study, DGI was found in 10% and it was more frequent in children treated with BPs (21%), than in those not treated (8%). Normally, DGI is less frequent in patients with OI type I compared with patients with OI type III/IV which is also demonstrated in the present study. The difference seen in patients with OI type I treated with BP compared with those not treated is most probably just a coincidence. Hypothetically, as teeth affected with DGI have more calcified tissue to be resorbed, this could delay tooth eruption. We found no difference in eruption between teeth with and without DGI.

Surprisingly, the eruption was significantly delayed in the healthy controls compared with the patients with OI type I treated with BPs. The high variation in number of erupted teeth in the healthy controls was probably a contributing factor. We found a delay in dental maturation in children treated before two years of age (Figs. 1 and 2) and as only three children with OI type I started BPs therapy before 2 years of age, the BP therapy was not reflected here.

In conclusion, BP therapy in OI patients seems to lower the dental age, and delay the dental maturity and tooth eruption. BP administration before 2 years of age might be a contributing factor.

### Limitations

In some cases, assessment of dental maturity was difficult due to either image quality or overlapping contours. Another limitation was the position of the cervical spine, which sometimes obscured the incisors in the radiographs, making these teeth difficult to assess. Unfortunately, the images were not taken under standardized conditions since the radiographs had been made using either screen-film or digital sensors in different clinics around Sweden over 27 years.
